# Preventing Atopic Diseases During Childhood – Early Exposure Matters

**DOI:** 10.3389/fimmu.2021.617731

**Published:** 2021-02-25

**Authors:** Mandy Pierau, Aditya Arra, Monika C. Brunner-Weinzierl

**Affiliations:** Department of Pediatrics, Medical Faculty, Otto-von-Guericke-University, Magdeburg, Germany

**Keywords:** immune tolerance, allergy prevention, children, regulatory T cells, allergen, Th2 cells, atopic disease, early exposure

## Abstract

Atopic diseases in childhood are a major burden worldwide and there is still a lack of knowledge about treatable causes. In industrialized countries such as Germany, almost every second child is sensitized to at least one common allergen. Recent studies show that although the predisposition to allergies is inherited, the adaptive immune system of neonates and infants follows a developmental trajectory and whether an allergy actually occurs depends also on timing of allergen exposure including diet as well as environmental factors. New recommendations are far from being rigid of allergen avoidance; it is rather moving toward conditions that stand for more biodiversity. The observation that introduction of peanuts or eggs early in life significantly reduced the development of a later allergy will change our recommendations for the introduction of complementary foods. This is consistent with the hygiene hypothesis that early provocation shapes the developing immune system so that it reacts appropriately. Therefore, promoting the development of tolerance is at the heart of sensible allergy prevention - and this begins with the last trimester of pregnancy. In light of this concept, actual recommendations are discussed.

## Introduction

Type I allergy (hypersensitivity) is caused by an aberrant production and activity of immunoglobulin E (IgE) against normally nonpathogenic antigens (called allergens). Atopic diseases are among the most common chronic health problems affecting children and young people all around the world (1). Although a large proportion of young children lose their reactions to food allergens, as they get older, many develop other types of allergic reactions, such as allergic dermatitis (AD), hay fever, asthma, or rhinitis. (2). The latter path of suffering is often described as the "allergic march" (2). A prerequisite for the development of allergy is a sensitization with the antigen and development of a memory response. Common antigenic allergens include animal dander, chemical additives, foods, insect bites, pollens, and even drugs. Upon antigen presentation to T helper cells, immune reaction is skewed toward type 2 (Th2) response, associated with cytokines Interleukin (IL)-4, IL-5, and IL-13 and leads to the production of IgE antibodies by B cells. These antibodies binds *via* the high-affinity IgE (FcεR1) receptors to mast cells or basophils (3). Reencounter with the same antigen causes cross-linking of the IgE/FcεR1 complex and an excessive allergen-specific IgE antibody production. Activated mast cells and basophiles degranulate and subsequently release mediators such as histamine and leukotrienes. Alongside with pathologically elevated allergen-specific IgE antibody titers these mediators contribute to typical signs and symptoms of atopic diseases and in severe cases, even anaphylactic shock may occur.

The definition of immune tolerance to allergens is establishment of a long-term clinical unresponsiveness to one specific or many allergens (4, 5). It is not just absence of a response, but also the suppression of the adverse allergic reaction. Enforcing and reinforcing tolerance has become an important goal for prevention and curative approaches for any atopic disease aiming for setting an optimal new response or correcting pre-existing dysregulated responses. In short, tolerance is induced against the identified allergen. The therapy of choice for inducing long-lasting tolerance is the allergen-specific immunotherapy (AIT), which is administered subcutaneously or sublingually. It is applicable for children as well as adults (6).

Sudden resolution of food allergy in infancy and childhood has been reported regularly. This natural induction of tolerance is often associated with reduction of allergen-specific antibody production of the IgE isotype and activity of basophils in addition to enhanced levels of allergen-specific antibodies of the IgG4 isotype and frequencies of regulatory T cells (Tregs) (7–9). This is in line with the finding that, successful AIT relates to enhanced numbers of Tregs and dendritic cells (DCs) as well as increased allergen-specific IgG4 levels. Thus, it seems plausible that the same immunological mechanisms involved in tolerance induction may drive protection from allergic disorders in early life. Therefore, promoting the development of tolerance is at the heart of sensible allergy prevention - and this begins with newborns, maybe even already during pregnancy.

Numerous recommendations for allergy prevention, as formulated in guidelines in Germany have been subject to considerable fluctuation on the basis of recently published clinical studies (10). In some areas a paradigm shift has taken place: In the past, "allergen avoidance" was the central principle of the recommendations around the prevention of allergies (11). In the meantime, it is progressively changing by moving toward an active "tolerance induction". This concept of tolerance induction is also important in term of pathophysiological considerations.

## Particularities of Atopic Diseases in Childhood

During infancy and childhood, atopic diseases are the most common chronic diseases (12). Current numbers from different countries show that up to 40 percent of children, and young people suffer from an atopic disease and the prevalence still increases (13). For example, the prevalence of asthma in Australian schoolchildren raised from 13% to 39% and from 23% to 44% for hay fever (14). In Denmark, the prevalence of AD enhanced from 17%–27% in 7 to 17-year-olds (15) and in Scotland from 5% to 12% (16). In 2010, African-American children in the U.S. were 5% more likely to have had allergic skin disorders than Caucasian or Asian children (17). In many developing countries, the incidence of allergies is on the rise. For example, the prevalence of eczema among South African youth raised from 12% to 19% (18). Even though allergies often start in childhood and can last a lifetime, there are some special features during childhood (19). Children are particularly sensitive to foreign substances, since the child's immune system faces a special challenge: on one hand, it should fend off pathogens, but on the other hand should not cause any collateral damage to the developing tissues. Already their skin barrier is different to the adults, it is much thinner and more permeable (20). Certain allergic diseases typically occur at a certain age, i.e., food allergies dominate in infants. Of note, 30% of food allergic children under the age of 18 are estimated to react to more than one food (21, 22). Reports show that this estimate rises to 70% in highly atopic children (23). In comparison to those with single food allergies, multiple food allergic children experience a greater decline in quality of life and suffer more often from nutritional deficiencies (24, 25). They are also less prone to spontaneously resolving their pre-existing allergies (26). The most common allergies are to cow's milk, eggs, peanuts, tree nuts, or wheat. Peanuts, tree nuts, fish, and shellfish commonly cause the most severe reactions. Therefore, parents should not, as often reported, stop offering this kind of food more and more, assuming there is no evidence of allergy. In toddlers and primary school children, however, allergic reactions are intensified by indoor allergens (cat allergens, house dust mites), but also by airborne allergens such as pollen (27). Adolescents mainly suffer from pollen allergies. The allergy risk is primarily determined by genetic factors. Children with at least one first-degree allergic relative such as parents and siblings are considered at risk of allergy (28). However, allergic diseases are also common in many children who do not belong to this risk groups. Besides genetic factors, various environmental conditions such as exhaust fumes or smoke play an important role in the development of allergic diseases (28, 29). Minimizing those factors that irritate the organism is crucial for the primary prevention of allergies. Although harmful environmental influences should be avoided, this is no longer recommended for the potential allergens themselves.

## Influence of Lifestyle During Pregnancy and Childhood on Allergy Sensitization

In industrial countries, one in four is suffering from allergy symptoms (13). The correlation of incidence with industrial countries is based on an urban lifestyle, which is associated with intensive hygiene and, consequently, low exposure to microbes such as bacteria, viruses, and fungi (30). Further epidemiological studies show an inverse correlation of hay fever, asthma and wheezing with microbial burden, pet ownership, family size, and infections. Animals in particular, and especially their diversity, are important for allergy protection. The resulting hygiene hypothesis states that early provocation by microbes shapes the developing immune system so that it reacts appropriately (30). Meaning that the immune response is directed against microbes, but not against foreign ones, although harmless environmental antigens. Regarding this, it was shown that resting T lymphocytes of newborns spontaneously activate their Th2 program, which is the basis of atopic reactions (31). This potentially allergic response is shut down if the lymphocytes are exposed to inflammatory inducing factors, as it would occur if provoked by microbes.

Multiple factors influence the development of neonatal immunity among them maternal IgG and cytokines, as well as antigen exposure and numbers of lymphocyte subpopulations and antigen presenting cells (APCs). A developmental trajectory of changes in cell composition of the blood has been demonstrated during the first seven days of life (32). Herein, the number of basophils, plasmacytoid DCs, natural killer cells, and neutrophils are decreased. In contrast, the number of myeloid DCs increased, while many other cell types remained constant. Furthermore, the serum concentration of the soluble immune markers chemokine CXCL10, IL-17A, macrophage-derived chemokine, and IFN-γ were enhanced. The chemokine CCL5, granulocyte colony stimulating factor 2, IL-10, and IL-6 decreased within the first seven days of life. Also other pure developmental features run early in life, like the creation of a diverse B-cell repertoire (33); maturation of the human fetal antibody repertoire requires five months, starting in the last trimester and according to gestational age, ending earliest at two months of age. Also natural IgM antibodies generated from the fetus against a defined set of autoantigens occurs assumed to protect certain structures from autoreactive T cells (34).

On top of that neonatal immunity shows an unambiguous bias toward production of Th2-cell polarizing cytokines (IL-4, IL-5, IL-10) - at least under some circumstances - and suboptimal Th1 cytokine production (IFN-γ, IL-2, and TNF-α) (35) and B cell differentiation as marginal zone B cells are hardly generated until two years of age (36, 37). The suppressed secretion of IFN-γ by Th1 cells was reported to be mediated by enhanced IL-4 transcription and production (38). The chromatin structure of the Th2 cytokines loci is hypo-methylated, which implies rapid transcription accounting for the bias toward Th2 type in neonatal CD4^+^ T cells (39). In terms of timing, investigations show that the modification of the CpG residues of the Th2 locus are found as early as fetus`s mid-gestation and lasts at least until the first week of life (40). Additionally, several studies reported that environmental co-stimulatory signals *in utero* affect differentiation of Th cell and mediate subpopulation characteristic profiles of genomes during immune system development (41). Therefore, timing is decisive in imprinting the immune system. However, when monitoring expression of CD40L by neonatal T cells, which needs to be upregulated for optimal T cell responses, its expression is potentially fully developed as shown by stimulation of neonatal CD4^+^ T cells with SEB (Staphylococcus Enterotoxin B) and monocytes or with *Candida albicans*-matured or *Staphylococcus aureus* (*S. aureus*) SEB^neg^-matured monocytes (42).

In terms of environmental toxicity, there are adverse effects of neonatal and infant exposure. With many developing organ systems toxic exposure of the fetus in the womb has even more serious consequences (43, 44). For example, exposure to cigarette smoke *in utero* effects the development of the immature lung and airways with additional impacts on the immune system.

Timing matters also for exposure to microbes. Many studies focus on the hygiene hypothesis postnatal but indeed, intrauterine exposure does already matter. The inclusion of pregnancy data in epidemiological studies on urban and farm environments and the child's immune system clearly showed that the child already reacts prenatally to stimuli: The exposure of the mother on the farm during pregnancy has a strong impact on the immune response of the child after birth (45). In addition, it has been shown that nutritional imbalance of the mother, deficiency as well as abundance, may have a significant impact on immunity in newborns and early life immune maturation (46). Therefore, immunomodulatory cells and cytokines containing human milk prevent respiratory infections as well as development of allergies in neonates and infants (47). *In utero*, the imprinting could start as early as the second trimester of pregnancy, because at this point the fetus starts to show first signs of immune competence. It is also shown that reinforced microbial contact during pregnancy is more powerful for prevention of allergies than starting at birth or even later (45). However, since the adaptive immune system, which is characterized by memory formation, develops mainly in the third trimester, the imprinting with a possible lifelong "memory" is more likely starting during this period.

## The Adaptive Immune System of Early Childhood

The postnatal phase is characterized by rapidly changing environmental and microbial exposures that stimulate immune development, requiring immune regulation to maintain homeostasis (48, 49). Managing such rapidly shifting, diverse functional requirements is at least partially achieved by compartmentalization (50). Analysis of T cells from lymphoid and mucosal tissue from pediatric organ donors in the first two years of life, revealed an early compartmentalization of T cell differentiation and regulation compared to adults. While memory T cells are mainly found in adult tissues, naïve recent thymic emigrants (RTEs) are the main subset in pediatric blood and tissues (50). RTEs are more similar to thymocytes than the mature naïve T cells and are far less abundant in adults (51). As neonatal Th cell compartments present unique properties contributing to the age-dependent balance of Th cell responses, we identified a novel CD31^+^ subset of naïve CD4^+^ T cells in infants adenoids (31). This subset expresses intracellularly an unglycosylated isoform of IL-4. These cells are not verifiable in adults and are suspected of being Th2 progenitor cells that are constrained to early age and spontaneously differentiate into mature IL-4 secreting Th2 cells. These cells have been associated with class switching for IgE production by B cells, although it has been shown for adults that T follicular helper cells secreting IL-4 support the class switch (52), very little is known about that in early life. In contrast to adult peripheral blood mononuclear cells, cord blood analysis show qualitative and quantitative variations in immune responses and distinct age-related immune responses are seen up to the age of 18 months, some also display up to 60 months after birth (42, 53, 54). Furthermore, study of cytokine co-expressing multifunctional CD4^+^ and CD8^+^ T cells showed that children three or older differed substantially from those aged one- or two-years, implying a developmental switch in differentiation of T cells at that age (55). However, like adults naïve T cells of neonates produce CD45RA isoforms, diversified T cell receptors and common co-stimulators at the cell surface such as CD27 and CD28.

Children growing up on farms are less prone to develop allergy, asthma or even leukemia later in life (56–58), stressing the point that exposing the immune system to bacteria and others within the first years of life leads to a conditioning that affects health and immunity later on. This can also be detrimental, i.e., it was shown that inhaling mold during early childhood did not cause acute clinical symptoms, rather was correlated to asthma development later on (59). Interestingly, the data from farm studies echo those from inner-city children studies. The URECA study is a birth cohort study launched in 2005 in economically deprived urban neighborhoods in inner-city Baltimore, Boston, New York City, and St Louis (60). The household environments of high-risk children starting from the prenatal period to the first three years of life were assessed in this study to determine modifiable risk factors for childhood asthma at seven years of age. Higher indoor/house dust levels of pet (mouse, cat) or pest (cockroach) allergens in infancy correlated with reduced incidence of asthma in high-risk urban children. In the first year of life, the bacterial microbiota in house dust differed between the homes of children who suffered from asthma or not. The excess of number of specific microbes were clearly related to either increased or decreased likelihood of developing asthma, and most of bacterial taxa correlated with the amounts of the indoor allergens associated with asthma risk. In addition, prenatal stress and exposure to tobacco smoke by the mother or depression scores in early life demonstrated to support development of asthma by the age of seven years. This suggests that an exposure to non-toxic environments early in life which contain potential allergens and microbes in abundance have been clearly correlated with reduced risk of asthma and other atopic diseases. Of note, it was demonstrated that the Th2-machinery is switched on in resting and suboptimal activated T cells of neonates unless they get inflammation signals (31). In addition, characteristic production of Th2 cytokines (IL-4, IL-5, IL-10) has been reported for T cells of term and pre-term born (41). Others used strong PMA and Ionomycin stimulation, and demonstrated an enhanced Th1-like cytokine expression (61). In older studies, Th1 responses have been shown to be evident but with a lower magnitude in pre-term and neonates when compared to adults (62). Others could show, that T cells of preterm and mature born neonates initiate far higher frequencies of IL-17 producers than adults upon polyclonal stimulation (63). Also IL-8 (CXCL8) was described to be a signatory cytokine, as already resting naïve T cells of neonates show IL-8 expression that is even further enhanced by polyclonal stimulation (64).

## Special Characteristics of Regulatory T Cells in Neonates

Recent study has shown that the ontogenesis of the immune system from fetus to adult does not proceed in a linear manner (65). Instead, it is stratified into layers of different immune cells that develop sequentially from distinct waves of hematopoietic stem cells (HSC). Therefore, T cells of neonates and adults T cells are discussed to have distinct origins. In contrast to T cells from adults, T cells of neonates originate from fetal HSC and display not only shorter T cell receptors (TCR), but also a more restricted T cell repertoire, in addition to an amplified peripheral homeostatic proliferation. Neonatal T cells rapidly differentiate into effector and Treg cells after activation than adult counterparts do, although at the cost of the formation of long-lived memory cells.

Tregs limit unnecessary immune responses, such as to strong Th2 responses that mediate allergic responses (66). They will be induced by bacterial or helminth infections among other things. Fetal naïve CD4^+^ T cells respond strongly to alloantigen`s, and tend to develop in presence of TGF-β toward Tregs, and thus actively promote self-tolerance (67). Peripheral Tregs account for ∼3% of total CD4^+^ T cells at birth (68) and these cells persist for an extended period of time (69). High numbers of Tregs - ∼12% of CD4^+^ T cells - exist in cord blood and ∼8% in neonatal lymph nodes (70, 71). Tregs account for a high proportion (30%–40%) of CD4^+^ T cells in pediatric tissues but only 1%–10% in adult tissues (50). They also showed that Tregs in human tissues exhibit distinct characteristics independent of age, influenced by CD45RA- phenotypes, together with tissue-specific signals. Furthermore, human neonatal non-differentiated naïve CD4^+^ T-cells demonstrate a TCR-dependent intrinsic default pathway to differentiate into Treg cells and exert suppressive functions (72). In neonates and infants, they represent an additional regulatory mechanism to prevent allergies and asthma because low numbers relate to enhanced risk to sensitize and develop AD early in life (31). Of note, a lower incidence of circulating Tregs at the time of birth correlates with unfavorable exposures, including maternal smoking and maternal allergy (73, 74). Others showed that Tregs from children are more effective when the mother had worked during pregnancy in a stable (75). Also exposure to non-processed milk had a significant impact leading to an increased number of Tregs in children aged four years, which in turn correlated with a reduced incidence of asthma and allergy (76).

## Recommendations for Allergy Prevention in Everyday Life

In the past, recommendations for protection against allergic diseases based on the belief that therapeutic strategies to avoid potent and major allergens are the best way to prevent allergy development. However, with this implementation prevalence of allergic diseases did not decrease but rather increased, likely reflecting a failure of immune tolerance mechanisms. There is mounting evidence that exposure to allergens, optimal in early years of life, is necessary for development of immune tolerance. The HealthNuts study showed that late introduction of egg did not decrease the risk of developing egg allergy (77). The study even suggest introducing cooked eggs between the age of 4–6 month and this may even protective against egg allergy.

Therefore, recommendations and guidelines, e.g., in Germany, changed completely. Furthermore, it is widely debated whether indoor allergens, such as HDM, furry pets, and fungi, are involved in the development of allergy (78), as results on their function as risk factors are often contradictory. Recently published data support a recent shift of prevention strategies from allergen avoidance to tolerance induction (79, 80). This concept of tolerance induction is also important in term of pathophysiological considerations.

Consequently, a number of recommendations have been "liberalized" in recent years, concerning early allergen elimination. HDM allergy is a key contributor to asthma therefore, it was questioned to which extend HDM elimination makes sense as a primary prevention measure. In the 1990s, a simple rule applied: Early exposure to allergens in infancy, e.g., HDM leads to a later allergic sensitization in a dose-dependent manner. On the contrary, results of large intervention studies have shown that although home renovation measures significantly reduced the indoor allergen exposure, these effects were not accompanied by a lower sensitization rate in children (81). For example, in the Manchester cohort (nacMAAS), in which 251 newborns with a positive family history of atopic diseases were followed up after a double-blind, placebo-controlled intervention with mite proof mattress covers, the intervention group showed a significant reduced mite allergen burden (82). However, the number of mite-sensitized children was significantly higher in the intervention group (23%) than in the control group (8.2%). Accordingly, such primary prevention of this kind cannot be recommended and is stated in several international guidelines. This does not, of course, extend to secondary and tertiary preventive measures, where the atopic disease is already established and strong proof of effectiveness remains.

**Figure 1:**
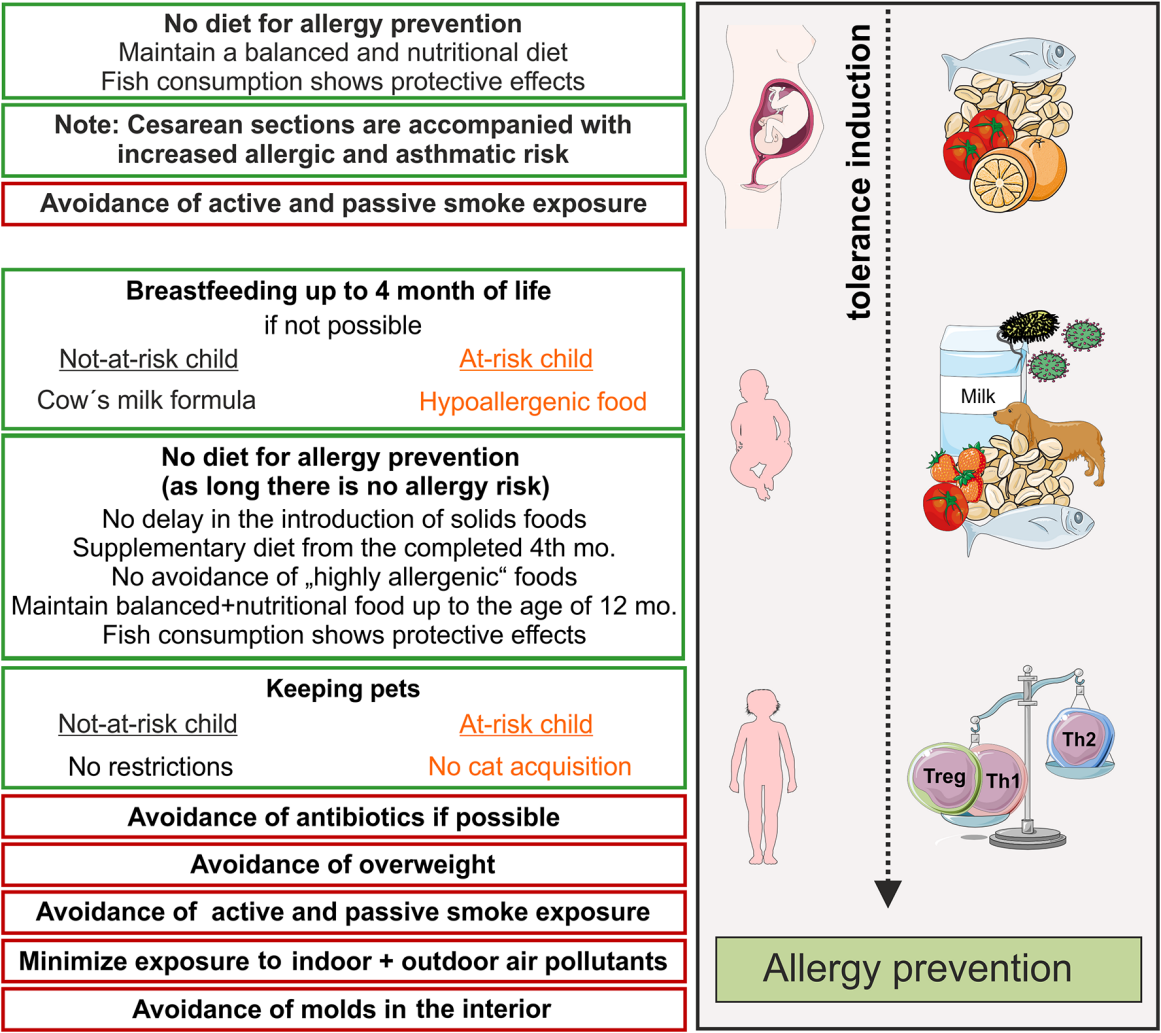
Primary prevention of allergic diseases (asthma, hay fever, atopic dermatitis). Contact to allergens (milk, peanut, fish, house dust mite) and microbes during pregnancy and early life leads to an active training of the immune system and, therefore, induces active immune tolerance. Thereby, allergy mediating T helper cell of type 2 (Th2)-answers are prevented and tolerance mediating regulatory T cells and Th1 cells dominate. Recommendations for primary allergy prevention during pregnancy and childhood. In orange shows recommendations for allergen avoidance in at–risk children, red boxes recommendations for avoidance of risk factors. Symbols are from smart servier medical art 2020 (https://smart.servier.com/). mo, month.

Recommendations concerning pets have also been liberalized and the principle of avoiding allergens has been increasingly abandoned. Therefore, data from 11 European birth cohorts were combined in a meta-analysis and published 2012. In this study, the pet keeping was differentiated with respect to the endpoints allergic sensitization, AD, food allergy, allergic rhinitis and asthma. Furthermore, they showed, that keeping dogs significantly reduced (by 28%) the risk of AD but non-significantly reduced the risk of asthma (83, 84). Keeping cats was also not associated with a likelihood of developing atopic diseases. Scattered studies however, indicate that keeping cats significantly enhanced the likelihood to develop AD especially when belonging to a at-risk group, e.g., when harboring a genetic alteration such as a loss-of-function mutation of filaggrin (85). For this reason, guidelines have been maintained for a stringent a recommendation for at-risk children, although the recommendations are phrased in a more user-oriented way. Therefore, the allergy prevention recommendations suggest that cats should never be purchased again in case of at-risk children. Nutrition can also contribute to the prevention of allergies. In recent years, avoidance of potential allergens during the first month of life has been considered as the best strategy for prevention of allergic diseases. Since the emergence of the hygiene hypothesis, however, this approach has been increasingly questioned (86). It contains that the risk for allergies increases if the immune system is not challenged. Many allergic mothers omit certain foods during pregnancy in concern that their child could also suffer from allergy. Nevertheless, there is no scientifically proven evidence that restricting food choices during pregnancy protects against the development of allergies during childhood. The same applies for the mother`s diet during breastfeeding, where antigens are detectable in breast milk and in very rare cases infants react allergic to these milk components. To date there is no evidence that avoiding potentially allergenic foods such as cow's milk, chicken egg or nuts has a preventive effect on the development of allergies (87). On the contrary, there is even evidence that fish consumption during pregnancy and breastfeeding has a protective influence on atopic disease development in children (88) (see below, **Figure 1**). Moreover, the benefits of restricting food choices must be weighed very critically against the risk of insufficient nutrient intake.

Furthermore, early introduction of food is beneficial but avoidance of food allergens early in life can be harmful because children especially with AD can become sensitized to food allergen by skin exposure. The International Study of Asthma and Allergies in Childhood (ISAAC) Phase III found that the prevalence of AD affects between 7% to 8% children (aged six to seven) and young teenager (aged 13 to 14) (89). Also up to 8% of children are affected from food allergy and its occurrence has been rising during the last 20 years (90). Although the origin of chronic inflammatory AD, which affects the skin, is not known, it is clear that a malfunction of the epithelial skin barrier is involved and subsequently leading to frequently inflamed skin. Due to its occurence early in infancy, it represents often the starting point of an atopic march. The severity of AD is a factor that determines the later development of an allergic disease. Prospective studies of birth cohorts have clearly related early-onset of AD with suffering from asthma and allergic rhinitis at school age. About 40% of AD patients develop a food allergy to peanut, egg, or sesame seed (91, 92).

The “dual-allergen exposure hypothesis” postulates that exposure to food allergens through the frequently inflamed skin in AD may cause allergic sensitization, ultimately provoking an allergy. Herein, the initial malfunctioning is the disrupted skin barrier leading to skin inflammation. In terms of acquiring immune tolerance to food allergens, it can be initiated early in life *via* oral exposure (93). Healthy skin is a natural barrier – physiological and immunological - not only against invasion of microbes, but also against penetration of environmental toxins and allergens into the body. Defects in the skin barrier can be due to several players among them malfunctioning of terminal epithelial differentiation, insufficiency of antimicrobial peptides (AMPs), modified stratum corneum intercellular lipids, dysbiosis of the skin microbiome and immune maladaptation (94). For epithelial barrier, genetic alterations encoding skin barrier proteins and unusual lipid production or tight junction formation add to its dysregulation and have been linked in AD to the development of food allergies. Today, there is increasing evidence that epicutaneous allergen sensitization is more likely to occur due to an impaired skin barrier (94, 95). It was demonstrated in an murine model using scraping of the skin that skin contact of potential allergens such as ovalbumin (OVA) or peanut proteins lead to the generation of IgE responses specific to egg and to peanut, respectively (96, 97). In another mouse model scratching was mimicked using epicutaneous sensitization with OVA on tape-stripes patched to the skin (98). The treatment conquered the immunological basis for a dramatic response when an additional oral challenge with OVA was given. Upon challenge, enhanced IgE-dependent intestinal mast cell expansion, enhanced concentrations of IL-4 in serum as well as systemic anaphylaxis were monitored. The assumption that the allergic march is causally supported by a malfunction of the skin barrier, which leads to a sensitization against food allergens and ultimately to food allergy, is confirmed from clinical studies. Studies show that newborns with elevated trans epidermal water loss (TEWL) are at a greater risk of having AD at one year of age (99) which was even predictive of developing food allergy in two-year-olds (100). Furthermore, when children in a birth cohort study were exposed to continuous low-dose allergen exposure in the form of peanut oil applied to inflamed skin sites, their risk of having peanut allergy at age five increased (101). The link between AD and food allergy became clear in the HealthNuts study, which found a 11 times higher incidence of peanut allergy in children who developed AD and an 6-fold enhanced risk to develop egg allergy by age one year (102). Usually, the skin barrier is supported by microbiota. A dysbiosis of it adds to an insufficient barrier function of the skin and ultimately to sensitization by epicutaneous allergens. Indeed, almost 90% of AD patients have a colonisation of *S. aureus* on their skin, which demonstrates clearly a disbiosis. The severity of the disease and its aggravations are associated with increased abundance of *S. aureus* and reduced local microbial diversity (103). Concluding, the dual allergen exposure hypothesis suggests that the decision whether the immune system is primed to develop allergy or tolerance depends on whether the first exposure to food allergens takes place through the skin or the gut during the first year of life. In addition, sensitization *via* the skin has been identified to trigger allergic reactions to food or allergic respiratory diseases even when the skin barrier is well intact. However, epicutaneous sensitization still needed an inflammatory event which was mimicked by adjuvant or highly concentrated continuous exposure of the antigen (104). Most of the key measures for prevention were aimed to reduce the risk of AD development by prophylactically protecting the skin barrier soon after birth in newborns with an elevated risk, e.g., supporting the skin by emollients from birth on which the incidence determined 6–8 months later indeed reduced (105, 106). Results of the study showed that the pH of the skin was lower in the emollient group and that the skin microbiome differed to the control group explaining the mechanism of preventative effects of emollients by regaining a "healthy" skin microbiome in high-risk infants (107). Several major studies are currently ongoing to investigate the effectiveness of regular moisturization in protecting the skin barrier from birth on not only to prevent AD development, but also challenge-tested atopic diseases such as food allergy and allergic asthma (108, 109). Attempts have been made to prove that probiotic supplementation during the fetal and infant period of life can prevent development of AD, but so far only little evidence is available (110). Besides some evidence for AD, prove is still missing at all for preventative effects of probiotic supplementation against food allergy or other atopic diseases.

Some randomized controlled studies addressing high-risk infants suffering from severe AD or already established food-sensitization, respectively, prevailed that early introduction of potential allergens in foods might limit the likelihood of developing allergies to peanuts or eggs. In the Prevention of Egg allergy with Tiny amount InTake (PETIT) study of Japanese high-risk infants with AD, development of egg allergy was demonstrated to reduce the risk by 30% compared with a control placebo group when egg powder was introduced early in life (111). Furthermore, orally reinforced aquisition of tolerance to egg allergy can be exploited therapeutically. In addition, a humanized monoclonal antibody (Omalizumab) against the Fc-part of IgE (anti-IgE) binds and neutralizes the IgE-type antibodies which are responsible for triggering the allergic reaction. It could become a preventative therapeutic strategy for solving food allergy, but also as a therapeutic complementation for triggering oral tolerance in egg allergy (112).

Peanut allergy has a potential anaphylactic risk and once acquired it is very difficult to outgrow. In the past some experts suggested that the introduction of foods that contain peanuts should be delayed until the three years of age (113), unfortunately this has contributed in part to a spike in peanut allergies over past few decades. The results of the in 2015 published LEAP ("Learning Early About Peanut") study received extensive publicity (114). The work based on the observation that Jewish children in England had a significantly higher prevalence of peanut allergy than children with the same genetic background living in Israel do. Unlike in England the children growing up in Israel had been exposed to peanut products much earlier. Based on this observation, a clinical intervention study has conducted, where 640 infants with severe eczema, egg allergy, or both consumed or avoided peanuts until 60 months of age. However, 321 instructed children and their families strictly avoided peanut-containing products and 319 children received 6 g peanut protein per week. Using a food provocation test at the age of five years, the prevalence of peanut allergy was significantly reduced in the consumption group (1.9%) compared to the avoidance group (13.7%; p<0.001). The findings of the LEAP study remained one year later in a follow-up study (115). There, all participants avoided any peanuts products for one year to determine whether the protective effect of early peanut intake last in the long term. The data indicate that the child's gastrointestinal tract may develop tolerance (meaning an adjusted intestinal barrier function and its secreted products, luminal digestion of antigens and a present suppressive immune milieu including Tregs (116)) rather than an allergic immune response in the first year of life through regular exposure to food. The work has led to numerous controversial discussions on the early introduction of highly allergenic foods into the complementary diet. Even if the results of the English group are speculative, it should first be taken into account that this is a very specific cohort before transferring it to other countries: only children participated with severe eczema and/or egg allergy. There are still some question marks, whether these results can be transferred to other countries and other populations and whether they are now mature enough to be used as practical recommendations for prevention. However, the National Institute of Allergy and Infectious Diseases (NIAID) in US already developed addendum guidelines for the prevention of peanut allergy, which displayed a significant paradigm shift in early food introduction (117). In order to minimize the risk of peanut allergy, they suggest, that the infants at highest risk (with severe eczema and/or egg allergy) should be introduced to age-appropriate foods containing peanuts as early as at the age of 4-6 months. These are the first guidelines that firmly recommend introduction of peanut-containing food to infants should start at approximately six month of age.

The mechanism behind the increase in food allergies over the past two decades is poorly understood. Therefore, various research focus on the investigation of the hygiene hypothesis, delivery by cesarean section, changes in dietary fat content, Vitamin D deficiency, or insufficiency, darker skin tone (118). Furthermore, late introduction to food proteins as well as exposure to more intact dietary proteins mediated by the usage of gastric acid suppressive medications (antacids) could be explanations for the rise in food allergies (119). In addition, food allergies are often associated with other allergic diseases such as eczema, family history of food allergy also plays a role, as well as immigration to developed counties during infancy, and genetic factor are being investigated (118, 120).

With the discussion about the early introduction of highly allergenic foods such as peanuts, the introduction of hydrolyzed infant formula for the prevention of allergic diseases was questioned. In the US and Europe the current recommendations are that at-risk infants should receive hydrolyzed infant formula if the mothers don´t, or only partly breastfeed them for the first 4 months (121, 122). Several meta-analysis (123, 124) or the German Infant Nutritional Intervention (GINI) study (125) found that high-risk infants who were fed with partially hydrolyzed whey formula or partially hydrolyzed formula showed a reduction of AD incidence and a decreased risk of any atopic manifestation. In contrast, a meta-analysis including 37 intervention studies as well as the Cochrane review published in 2018 showing no consistent evidence that partially or extensively hydrolyzed infant formula reduce the risk of allergic diseases in at-risk children (126, 127). The Australasian Society of Clinical Immunology and Allergy (ASCIA; www.allergy.org.au) already adjusted the current guidelines accordingly and recommend using commercial infant formula until the age of 12 months if an infant is not or only partially breastfeed. However, since different hydrolyzed formulas exist, the allergenicity, tolerance, efficacy and clinical impact also differ. Therefore, every product should be tested. The effects of the routine use of the different hydrolyzed formulas in larger populations of non-exclusively breastfed infants need further investigation to determine the benefits. Adaptation of food for allergy prevention aims, among other things, at an "optimal" microbiome in the gut. However, if confrontations of the organism with pathogenic bacteria are prevented early in life, the risk of asthma and allergies increases (128, 129). For example, administration of antibiotics in the first 6 months of life, increases the incidence of atopic diseases at the age of six (54). Pasteurization of cow's milk kills bacteria. However, the drinking of unprocessed milk during pregnancy protects from allergies and asthma development (129). These examples also show that avoiding allergens is not the best way to prevent allergies.

## Probiotics, Prebiotics, and Bacterial Lysates

The above-mentioned examples clearly show in which direction changes in current allergy prevention are going: Away from rigid recommendations to avoid allergens, both in the area of nutrition and pet keeping, and toward environmental conditions that stand for more biodiversity. Therefore, the so-called farm studies fit perfectly into this concept. The early exposure to farm animals in the first year of life and the consumption of unprocessed cow's milk had a significant protective effect on the development of allergic sensitization, allergic rhino conjunctivitis and bronchial asthma (130). Noteworthy, in this context epidemiological data indicate a higher risk of developing asthma after a caesarean section (131). Similar, others observed a reduced risk of allergic diseases in families with an anthroposophical lifestyle, which was characterized, among other things, by a lower use of antibiotics (132).

Allergy preventive recommendations become more and more enumerations about which measures do not show any allergy preventing effects (avoidance of pet keeping) or which measures are to be omitted (caesarean section, early antibiotic administration) but in the end these measures do not actively induce tolerance (**Figure 1**). Therefore, it would be desirable to have recommendations based on interventions that actively promote tolerance induction.

From the farm studies, it has become clear, that the microbiota plays a key role promoting tolerance early in life. During the first three years, the diversity of the gut microbiome is built up. Lack of microbial seeding at birth by cesarean section was proposed as a cause that influence the increase in asthma and allergic diseases (133). During labor and vaginal delivery, the neonate acquires the vaginal and fecal microbiota of the mother. This microbial exposure also helps to rebalance the neonate's immune system that has a larger fraction of Tregs and a higher Th2:Th1 ratio. Results from an Australian study with nearly 500,000 participants shows, that neonates born by emergency cesarean section had a higher rate of metabolic disorders, such as obesity or diabetes (134). However, there was no substantial correlation between cesarean section and the incidence of allergic diseases in other findings (135). Variations in the results could be due to factors that may mediate or change the impact of microbial seeding. For instance, as already discussed, breastfeeding has a protective influence on the early onset of allergic diseases such as asthma, AD and food allergies (136). Breast milk contains beneficial microbes that are transmitted from mother to child (137) and one of the first bacteria is *Bifidobacterium longum* (*B. longum*) which is colonizing the gut by a fast expansion. *B. longum* is an anaerobic, Gram-positive microorganism, which is considered to be a beneficial commensal (138). It is assumed that it spreads rapidly in the intestine to function as a placeholder to outcompete colonization of aggressive bacteria such as *S. aureus*. Of note, *S. aureus* is the most important example of a commensal bacteria becoming an aggressive antibiotic-resistant pathogen (139). In a mouse model for atopic airway disease, *B. longum* has been shown to induce Tregs which correlates with reduced airway atopic reactions (140). How *B. longum* mechanistically impacts on the human T cell responses is not known, so far. Also other beneficially acting bacteria such as Lactobacillus have been addressed (141).

As already mentioned, if confrontations of the organism with pathogenic bacteria are prevented early in life, the risk of asthma and allergies increases (128, 129). This assumption was investigated in a randomized trial in which bacterial lysates were administered orally to approximately 600 infants with at least one parent suffering from allergy (142). The applied lysates, which were given during the first two decades of life, were of heat‐killed *Escherichia coli* and *Enterococcus faecalis* or just placebo. At school age, neither development of AD, asthma nor allergic rhinitis were affected.

Accumulating data on this subject have led to the concept that an early administration of probiotics, prebiotics or bacterial lysates might be beneficial. Despite a large number of clinical studies, especially on the administration of probiotics, the data available are not yet sufficient to make generally valid recommendations. In some studies, effects of probiotic administration on atopic eczema have been observed, but these cannot be reproduced with identical preparations in other populations (143, 144). There might be protective effects in subgroups, like for children after caesarean section. Apparently, it is necessary to administer them during pregnancy in order to achieve preventive effects (145). In a meta-analysis, 29 studies on probiotic administration were summarized and revealed a 29% reduction of the eczema risk if supplementation was already given during pregnancy. When administered exclusively postnatal, this effect was lower with a 17% reduction and no longer statistically significant. Protective effects of primary prevention with probiotics on asthma, allergic rhino conjunctivitis or allergic sensitization were not observed.

Another meta-analysis assessed the impact of supplementation of prebiotics in infants as well as pregnant and breastfeeding women (146). In summary, they find insufficient proof to substantiate or refute a protective or a detrimental effect from randomized or observational studies, but cautiously suggest that prebiotics may minimize the likelihood of persistent wheezing in infants. Nevertheless, the authors claim that the safety on these suggestions is indeed limited because of the possibility of bias, indirectness of the evidence, and the inaccuracy due to small number of events of the estimated results. Two more clinical trials were also performed in addition to this meta-analysis (147, 148). One study reported early-life nutrition with prebiotics had an impact on the structure of the infant intestinal microbiota with a possible correlation between microbiota activity and the development of eczema in early life (147). Data published on a study addressing supplementation with galacto-oligosaccharide/polydextrose (GOS/PDX) for 48 weeks of high-atopy-risk infants due to parental history of allergic diseases did not show an impact on AD up to 96 weeks of age (148). However, in infants with GOS/PDX supplementation the incidence of respiratory infections up to 48 weeks was smaller than in infants fed with standard formula (p = 0.023), but was detectable for not more than 96 weeks. The colonization with Bifidobacterium and Clostridium cluster I increased over time in the GOS/PDX supplemented group. Bifidobacterium had a protective effect in respiratory infection; whereas Clostridium cluster I was linked with atopy protection.

In summary, there is still insufficient compelling data from human infant studies to support the beneficial impact of prebiotic supplementation for prevention of allergies. Also triggering the intestinal immune system with bacterial lysates does not show prevention of AD. Thus, the allergy preventive effects of intestinal colonization and immune modulation mediated by bacterial compounds early in life are by far not yet understood.

## Omega-3 Fatty Acid and Personalized Allergy Prevention

Studies performed to date indicate that higher intake of fish, sources of long chain omega-3 (n-3) polyunsaturated fatty acids (PUFA), during pregnancy is associated with lower risk of allergy development in infants and children (149). A Finnish study reported that a reduced intake of n-3 PUFAs during pregnancy increases the chances of developing asthma in children aged five years (150). Interestingly, children suffering from allergic diseases often had lower than normal amounts of the n-3 PUFAs EPA (eicosapentaenoic acid) and DHA (docosahexaenoic acid) in the cord blood (151). Furthermore, studies have reported that supplying pregnant women with fish oil is linked to immunologic changes in cord blood (152). These immunological effects might modify allergic sensitization and the risk to develop allergic diseases. Additional, since 16-year olds showed a substantial reduction in asthma-related diagnosis when their mothers were fish oil supplemented during late pregnancy, long term consequences of any immunological changes were suggested to have occurred in pregnancy and early life. A large standardized double-blinded placebo-controlled investigation found that intake of fish oil during pregnancy significantly reduces recurrent wheeze and asthma in 3- to 5-year olds children (153), whereby a total of 136 of 695 children (19.6%) showed these symptoms. The risk was significantly lower in the intervention group with 16.9% than in the placebo group with 23.7% (p = 0.035). One interesting finding was that the protective impact of maternal EPA plus DHA on children with recurrent wheezing or asthma was observed predominantly in the subgroup of children whose mothers had the lowest blood levels of these two omega-3 fatty acids at the beginning of the study. This indication could point toward a more personalized strategy of allergy prevention in the future. Similar approaches of personalized prevention, which stratify children into different risk groups, would be desirable for future work.

Taken together, early exposure to the n-3 PUFAs in pregnancy can be a strategy to induce immune effects that prevent infant and childhood allergic disease. Nevertheless, the available data are not completely consistent. In order to characterize more precisely the immunological and clinical effects and to evaluate their protective effects and persistence, further studies on the increased supply of long chain n-3 PUFA during pregnancy, and infancy are required.

## Conclusion

Just how to prevent atopic diseases in childhood, is a "billion dollar" question as it may be prevention for a lifetime. On the one hand, during the initial allergen exposure, especially the prevailing environmental conditions have a great influence by modifying the immune responses. Therefore, as a logical consequence, pregnant women and parents of infants are advised to limit risk factors. These include smoking, pollutants, and any unfavorable proinflammatory exposures. On the other hand, it includes a balanced and nutritional diet. To achieve more tolerogenic conditions during pregnancy and early life is at the heart of sensible allergy prevention and there is growing evidence that exposure to allergens is necessary for development of immune tolerance. According to latest studies administering antibiotics too early or the usage of hydrolyzed cow´s milk formula does not prevent allergic disease in high at-risk infants. Similarly, the observation that the early introduction of peanut-containing foods could significantly reduce the development of a later peanut allergy in British children will change recommendations for the introduction of complementary foods. The recommendations on breastfeeding over the first four months, avoiding environmental tobacco smoke, and avoidance of overweight will remain unchanged. Studies from tolerance induction in established allergic disease underline the significance of Treg cells. Optimizing the environment especially during the last trimester of pregnancy and first months of life to favour these regulatory pathways represents a good strategy to prevent allergy development. However, knowledge about induction and functioning of neonatal and pediatric Treg cells are scarce. As many of the approaches would have broader biological impact, improved understanding of early life immune responses combined with determinants of atopic diseases will be of interest. This offers a broader perspective for strategies to prevent allergic disorders such as early administration of probiotics, prebiotics, bacterial lysates, but also polyunsaturated omega-3 oils and nutrients with anti-inflammatory properties.

The precise time window of possible successful interventions for allergic protection must still be determined and mechanistically understood. Knowledge of this timeframe and the mechanisms for implementing tolerance to potential allergens could make it possible to guide the immune system in a short, defined time interval with anti-inflammatory nutrients. Thus, a much better understanding of the functioning of the neonatal and pediatric immune system is necessary to improve measures to prevent atopic disease.

## Author Contributions

MB-W, MP, and AA wrote the manuscript. All authors read the manuscript and provided important intellectual input to the manuscript. The corresponding author had full access to the data and had final responsibility for the decision to submit for publication. All authors contributed to the article and approved the submitted version.

## Funding

The work was supported by DFG Br 1860/12-1.

## Conflict of Interest

The authors declare that the research was conducted in the absence of any commercial or financial relationships that could be construed as a potential conflict of interest.
